# Bias in error estimation when using cross-validation for model selection

**DOI:** 10.1186/1471-2105-7-91

**Published:** 2006-02-23

**Authors:** Sudhir Varma, Richard Simon

**Affiliations:** 1 Biometric Research Branch, National Cancer Institute, Bethesda MD, USA

## Abstract

**Background:**

Cross-validation (CV) is an effective method for estimating the prediction error of a classifier. Some recent articles have proposed methods for optimizing classifiers by choosing classifier parameter values that minimize the CV error estimate. We have evaluated the validity of using the CV error estimate of the optimized classifier as an estimate of the true error expected on independent data.

**Results:**

We used CV to optimize the classification parameters for two kinds of classifiers; Shrunken Centroids and Support Vector Machines (SVM). Random training datasets were created, with no difference in the distribution of the features between the two classes. Using these "null" datasets, we selected classifier parameter values that minimized the CV error estimate. 10-fold CV was used for Shrunken Centroids while Leave-One-Out-CV (LOOCV) was used for the SVM. Independent test data was created to estimate the true error. With "null" and "non null" (with differential expression between the classes) data, we also tested a nested CV procedure, where an inner CV loop is used to perform the tuning of the parameters while an outer CV is used to compute an estimate of the error.

The CV error estimate for the classifier with the optimal parameters was found to be a substantially biased estimate of the true error that the classifier would incur on independent data. Even though there is no real difference between the two classes for the "null" datasets, the CV error estimate for the Shrunken Centroid with the optimal parameters was less than 30% on 18.5% of simulated training data-sets. For SVM with optimal parameters the estimated error rate was less than 30% on 38% of "null" data-sets. Performance of the optimized classifiers on the independent test set was no better than chance.

The nested CV procedure reduces the bias considerably and gives an estimate of the error that is very close to that obtained on the independent testing set for both Shrunken Centroids and SVM classifiers for "null" and "non-null" data distributions.

**Conclusion:**

We show that using CV to compute an error estimate for a classifier that has itself been tuned using CV gives a significantly biased estimate of the true error. Proper use of CV for estimating true error of a classifier developed using a well defined algorithm requires that all steps of the algorithm, including classifier parameter tuning, be repeated in each CV loop. A nested CV procedure provides an almost unbiased estimate of the true error.

## Background

The unique characteristics of microarray data have stimulated the development of a multitude of analysis methods. Microarray data is distinguished by very small numbers of samples compared to the number of features measured. Most previous machine learning methods have been developed on data where the opposite holds true; the number of samples is much larger than the number of features. As a result, such analysis methods have to be modified for microarray datasets.

An example is the common paradigm of splitting the data-set into training and test data. The training data is used for selecting features and training a classifier. Once a final classifier has been specified, it can be used to predict the classes of the test samples. The mean error on a sufficiently large (ideally infinite) test dataset gives the *true error *of the classifier.

When the number of samples *n *is small, it is important to ensure that the data used to test the classifier is not part of the data used to train it. Testing the classifier on the same samples that were used to train it gives the *re-substitution estimate *of the true error, which is known to give falsely low (usually zero) error estimates for small *n*.

With microarray data, splitting the sample into large training and test sets is usually not feasible since the number of samples is so small. Cross-validation (CV) is one solution to the lack of sufficiently large training and testing sets [1], where, instead of testing a fixed classifier (as we had in the split sample case) we have a fixed *classifier training algorithm*. A classifier training algorithm takes a set of samples and does feature selection and classifier training and returns a single, well defined classifier. In CV, part of the data is left out and the rest is used by the classifier training algorithm to develop a classifier. The classifier thus obtained is used to predict the classes of the left out samples. This loop is repeated for different left out portions. The average error thus obtained on the entire dataset (the *CV error estimate*) can be interpreted as an estimate of the true error for the classifier we would obtain if we used the classifier training algorithm on the entire dataset. In the case where the left out data consists of one sample only (Leave-One-Out-CV), it can be shown that the CV error estimate is an almost unbiased estimate of the true error expected on an independent test set for the classifier one would obtain if the classifier training algorithm was used on the entire dataset (Theorem 10.8, 8).

However, CV methods are proven to be unbiased only if all the various aspects of classifier training takes place inside the CV loop. This means that all aspects of training a classifier *e.g*. feature selection, classifier type selection and classifier parameter tuning takes place on the data not left out during each CV loop. It has been shown that violating this principle in some ways can result in very biased estimates of the true error. One way is to use all of the training data to choose the genes that discriminate between the two classes and only change the classifier parameters inside the CV loop. This violates the principle that feature selection must be done for each loop separately, on the data that is not left out. As pointed out by Simon *et al*. [2], Ambroise and McLachlan [3] and Reunanen [4], this gives a very biased estimate of the true error; not much better than the resubstitution estimate. Over-optimistic estimates of error close to zero are obtained, even for data where there is no real difference between the two classes.

Another violation of the principle is to do any kind of classifier parameter selection outside the CV loop. Examples of these classifier parameters are the numbers of neighbors for a Nearest Neighbor classifier or kernel parameters for the Support Vector Machine (SVM) classifier. To find the best values of these parameters for a given dataset, we can compute the CV error estimate for the dataset using different values of the parameters. Then the classifier parameter with the minimum CV error estimate is chosen to create the final classifier. The final classifier is trained on the entire dataset using the chosen optimal classifier parameters.

This comes under the general term of *wrapper methods*, where a CV algorithm is "wrapped" inside a search algorithm that tries to minimize the CV error. Such wrapper methods have proven very useful for data-driven adaptation of classifier parameters.

However, this involves a kind of additional training of the classifier (in the form of selecting the classifier parameter) that is done outside the CV loop. This violates the assumption that all training is done within the CV loop on the data not left out. Thus the guarantee of unbiased estimation of true error is not valid and there is a possibility of bias. In other words, the CV error estimate for the classifier parameters that minimize the CV error estimate could be a biased estimate of the true error of the final classifier trained on all the data using the optimal classifier parameters.

We investigate this possibility using two wrapper algorithms. The first is the Shrunken Centroids method of Tibshirani *et al*. [5] where an optimum value of a classifier parameter Δ that controls the degree of shrinkage is obtained as the one that minimizes the 10-fold CV error. The second is a variant of the Support Vector Machine proposed by Peng *et al*. [6] which selects SVM kernel parameters that minimize the Leave-One-Out-CV (LOOCV) error. The first article uses both the CV error estimate on the training set and the error on the test set for determining the optimal value of the parameters, thus making the test set part of the training process. The second article presents only the minimum CV error estimate obtained on the training set. The true error obtained on an independent test set is not given for either.

Since selection of classifier parameters that minimize CV error estimates is a kind of training, it should be included as part of the classifier training algorithm. Thus the classifier training algorithm in this case is the complete *wrapper algorithm *where, given a dataset, the classifier is trained the following way. First the CV error estimate is computed for different values of the classifier tuning parameters. Then, the parameters with the smallest CV error estimate are used to create a classifier using all the data. This satisfies the definition of a classifier training algorithm, *i.e*. an algorithm that takes a dataset and returns a single, well defined classifier.

Now that we have a wrapper algorithm that is a well defined classifier training algorithm, we can use CV to get an estimate of the true error for the classifier it returns. We can embed the complete wrapper algorithm (with its own CV loop for finding the best classifier parameters) inside another CV loop that computes the error estimate. Note that this is no different from the usual CV method. Here, instead of using CV to find an error estimate for a particular classifier (*e.g*. Nearest Neighbors) we use CV to find an error estimate for an optimized classifier (*e.g*. Nearest Neighbors with the optimal number of neighboring samples determined by minimizing the CV error estimate). Thus there are two CV loops; the inner loop is part of the wrapper algorithm and the outer loop computes an estimate of the true error. A similar method was used by Izuka *et al*. [7]. In this article, we investigate the effect on the bias when using this *nested CV *approach.

### Shrunken centroids

This classifier, originally proposed by Tibshirani *et al *[5], is an extension of the nearest centroid classifier. In the nearest centroid classifier the training set is used to calculate the centroids x¯1
 MathType@MTEF@5@5@+=feaafiart1ev1aaatCvAUfKttLearuWrP9MDH5MBPbIqV92AaeXatLxBI9gBaebbnrfifHhDYfgasaacH8akY=wiFfYdH8Gipec8Eeeu0xXdbba9frFj0=OqFfea0dXdd9vqai=hGuQ8kuc9pgc9s8qqaq=dirpe0xb9q8qiLsFr0=vr0=vr0dc8meaabaqaciaacaGaaeqabaqabeGadaaakeaaieqacuWF4baEgaqeamaaBaaaleaacqaIXaqmaeqaaaaa@2F5F@ and x¯2
 MathType@MTEF@5@5@+=feaafiart1ev1aaatCvAUfKttLearuWrP9MDH5MBPbIqV92AaeXatLxBI9gBaebbnrfifHhDYfgasaacH8akY=wiFfYdH8Gipec8Eeeu0xXdbba9frFj0=OqFfea0dXdd9vqai=hGuQ8kuc9pgc9s8qqaq=dirpe0xb9q8qiLsFr0=vr0=vr0dc8meaabaqaciaacaGaaeqabaqabeGadaaakeaaieqacuWF4baEgaqeamaaBaaaleaacqaIYaGmaeqaaaaa@2F61@ (mean expressions of the genes) for the two classes. A new sample is compared to the two centroids and classified according to the class of the nearest centroid. In the shrunken centroids method, a parameter Δ is used to shrink the class centroids towards the overall centroid after standardizing by the within class standard deviation. The centroid belonging to class *k *is brought closer to the overall centroid x¯
 MathType@MTEF@5@5@+=feaafiart1ev1aaatCvAUfKttLearuWrP9MDH5MBPbIqV92AaeXatLxBI9gBaebbnrfifHhDYfgasaacH8akY=wiFfYdH8Gipec8Eeeu0xXdbba9frFj0=OqFfea0dXdd9vqai=hGuQ8kuc9pgc9s8qqaq=dirpe0xb9q8qiLsFr0=vr0=vr0dc8meaabaqaciaacaGaaeqabaqabeGadaaakeaacuWG4baEgaqeaaaa@2E3D@ (mean of samples of all classes pooled together) by

x¯k=x¯+mk(s+s0)sign(dk)(|dk|−Δ)+     (1)
 MathType@MTEF@5@5@+=feaafiart1ev1aaatCvAUfKttLearuWrP9MDH5MBPbIqV92AaeXatLxBI9gBaebbnrfifHhDYfgasaacH8akY=wiFfYdH8Gipec8Eeeu0xXdbba9frFj0=OqFfea0dXdd9vqai=hGuQ8kuc9pgc9s8qqaq=dirpe0xb9q8qiLsFr0=vr0=vr0dc8meaabaqaciaacaGaaeqabaqabeGadaaakeaaieqacuWF4baEgaqeamaaBaaaleaacqWGRbWAaeqaaOGaeyypa0Jaf8hEaGNbaebacqGHRaWkcqWFTbqBdaWgaaWcbaGaem4AaSgabeaakmaabmaabaGae83CamNaey4kaSIaem4Cam3aaSbaaSqaaiabicdaWaqabaaakiaawIcacaGLPaaacqqGZbWCcqqGPbqAcqqGNbWzcqqGUbGBcqGGOaakcqWFKbazdaWgaaWcbaGaem4AaSgabeaakiabcMcaPmaabmaabaWaaqWaaeaacqWFKbazdaWgaaWcbaGaem4AaSgabeaaaOGaay5bSlaawIa7aiabgkHiTiabfs5aebGaayjkaiaawMcaamaaBaaaleaacqGHRaWkaeqaaOGaaCzcaiaaxMaadaqadaqaaiabigdaXaGaayjkaiaawMcaaaaa@5571@

where **s **is the vector of pooled within class standard deviation for all genes, *s*_0 _is the median of the elements of **s**, **d**_*k *_is given by

dk=x¯k−x¯mk(s+s0)     (2)
 MathType@MTEF@5@5@+=feaafiart1ev1aaatCvAUfKttLearuWrP9MDH5MBPbIqV92AaeXatLxBI9gBaebbnrfifHhDYfgasaacH8akY=wiFfYdH8Gipec8Eeeu0xXdbba9frFj0=OqFfea0dXdd9vqai=hGuQ8kuc9pgc9s8qqaq=dirpe0xb9q8qiLsFr0=vr0=vr0dc8meaabaqaciaacaGaaeqabaqabeGadaaakeaaieqacqWFKbazdaWgaaWcbaGaem4AaSgabeaakiabg2da9maalaaabaGaf8hEaGNbaebadaWgaaWcbaGaem4AaSgabeaakiabgkHiTiqb=Hha4zaaraaabaGae8xBa02aaSbaaSqaaiabdUgaRbqabaGcdaqadaqaaiab=nhaZjabgUcaRiabdohaZnaaBaaaleaacqaIWaamaeqaaaGccaGLOaGaayzkaaaaaiaaxMaacaWLjaWaaeWaaeaacqaIYaGmaiaawIcacaGLPaaaaaa@4366@

and (...)_+ _denotes the positive part of the quantity in the parenthesis, *i.e*. equal to the quantity if it is greater than zero, and zero otherwise. Thus genes which are not very differentially expressed will contribute less to the classification than genes that are more discriminating. The parameter Δ can be varied to vary the number of genes used.

In the original paper, 10-fold CV is used to obtain the CV error estimate for a particular choice of Δ. There is no objective guideline given for selecting Δ based only on the training data. The authors vary Δ and use the value that minimizes the CV error estimate on the training set and the error on the testing data simultaneously. Thus the test set is used in the selection of the classifier parameters, which is problematic.

### Support Vector Machines

Peng *et al*. present a method for feature selection using genetic algorithms (GA) and recursive feature elimination (RFE) in combination with a support vector machine (SVM) classifier [6]. SVMs were introduced by Vapnik [8] as linear hyperplanes that separate data belonging to different classes while maximizing the *margin*, or the distance of the training samples to the linear separating hyperplane.

Denote the two classes by 1 and -1. For a sample **x **consisting of *p *measurements (*e.g*. gene expressions) the *linear hyperplane classifier c*(**x**) predicts the class according to

c(x)={1if x^w′≥0−1if x^w′<0     (3)
 MathType@MTEF@5@5@+=feaafiart1ev1aaatCvAUfKttLearuWrP9MDH5MBPbIqV92AaeXatLxBI9gBaebbnrfifHhDYfgasaacH8akY=wiFfYdH8Gipec8Eeeu0xXdbba9frFj0=OqFfea0dXdd9vqai=hGuQ8kuc9pgc9s8qqaq=dirpe0xb9q8qiLsFr0=vr0=vr0dc8meaabaqaciaacaGaaeqabaqabeGadaaakeaacqWGJbWycqGGOaakcqWG4baEcqGGPaqkcqGH9aqpdaGabaqaauaabeqaciaaaeaacqaIXaqmaeaacqqGPbqAcqqGMbGzcqqGGaaiieqacuWF4baEgaqcaiqb=Dha3zaafaGaeyyzImRaeGimaadabaGaeyOeI0IaeGymaedabaGaeeyAaKMaeeOzayMaeeiiaaIaf8hEaGNbaKaacuWF3bWDgaqbaiabgYda8iabicdaWaaaaiaawUhaaiaaxMaacaWLjaWaaeWaaeaacqaIZaWmaiaawIcacaGLPaaaaaa@4B82@

for a weight vector **w **= ⌊*w*_0 _*w*_1 _... *w*_*p*_⌋ and the augmented sample vector x^
 MathType@MTEF@5@5@+=feaafiart1ev1aaatCvAUfKttLearuWrP9MDH5MBPbIqV92AaeXatLxBI9gBaebbnrfifHhDYfgasaacH8akY=wiFfYdH8Gipec8Eeeu0xXdbba9frFj0=OqFfea0dXdd9vqai=hGuQ8kuc9pgc9s8qqaq=dirpe0xb9q8qiLsFr0=vr0=vr0dc8meaabaqaciaacaGaaeqabaqabeGadaaakeaaieqacuWF4baEgaqcaaaa@2E3B@ obtained by appending sample **x **with a constant 1, *i.e*. x^
 MathType@MTEF@5@5@+=feaafiart1ev1aaatCvAUfKttLearuWrP9MDH5MBPbIqV92AaeXatLxBI9gBaebbnrfifHhDYfgasaacH8akY=wiFfYdH8Gipec8Eeeu0xXdbba9frFj0=OqFfea0dXdd9vqai=hGuQ8kuc9pgc9s8qqaq=dirpe0xb9q8qiLsFr0=vr0=vr0dc8meaabaqaciaacaGaaeqabaqabeGadaaakeaaieqacuWF4baEgaqcaaaa@2E3B@ = ⌊1, *x*_1_, ..., *x*_*p*_⌋.

The *margin *of a sample **x **of class *y *∈ {-1,1} is defined as *y*x^
 MathType@MTEF@5@5@+=feaafiart1ev1aaatCvAUfKttLearuWrP9MDH5MBPbIqV92AaeXatLxBI9gBaebbnrfifHhDYfgasaacH8akY=wiFfYdH8Gipec8Eeeu0xXdbba9frFj0=OqFfea0dXdd9vqai=hGuQ8kuc9pgc9s8qqaq=dirpe0xb9q8qiLsFr0=vr0=vr0dc8meaabaqaciaacaGaaeqabaqabeGadaaakeaaieqacuWF4baEgaqcaaaa@2E3B@**w' **and it plays a very important role in SVM. The margin of correctly classified samples is positive and that for misclassified samples is negative. The margin of the classifier is defined as the smallest margin of all the training samples. The SVM tries to find a **w **such that the margin is maximized while the norm of the weight vector, ⟨**w**, **w**⟩ is minimized. This is equivalent to minimizing the cost function

Φ(w)=12〈w,w〉+C∑i=1nξi(w)     (4)
 MathType@MTEF@5@5@+=feaafiart1ev1aaatCvAUfKttLearuWrP9MDH5MBPbIqV92AaeXatLxBI9gBaebbnrfifHhDYfgasaacH8akY=wiFfYdH8Gipec8Eeeu0xXdbba9frFj0=OqFfea0dXdd9vqai=hGuQ8kuc9pgc9s8qqaq=dirpe0xb9q8qiLsFr0=vr0=vr0dc8meaabaqaciaacaGaaeqabaqabeGadaaakeaacqqHMoGrcqGGOaakieqacqWF3bWDcqGGPaqkcqGH9aqpdaWcaaqaaiabigdaXaqaaiabikdaYaaadaaadaqaaiab=Dha3jabcYcaSiab=Dha3bGaayzkJiaawQYiaiabgUcaRiabdoeadnaaqahabaacciGae4NVdG3aaSbaaSqaaiabdMgaPbqabaGccqGGOaakcqWF3bWDcqGGPaqkaSqaaiabdMgaPjabg2da9iabigdaXaqaaiabd6gaUbqdcqGHris5aOGaaCzcaiaaxMaadaqadaqaaiabisda0aGaayjkaiaawMcaaaaa@4D18@

where

ξ_*i *_(**w**) = (1 - *y*_*i*_x^i
 MathType@MTEF@5@5@+=feaafiart1ev1aaatCvAUfKttLearuWrP9MDH5MBPbIqV92AaeXatLxBI9gBaebbnrfifHhDYfgasaacH8akY=wiFfYdH8Gipec8Eeeu0xXdbba9frFj0=OqFfea0dXdd9vqai=hGuQ8kuc9pgc9s8qqaq=dirpe0xb9q8qiLsFr0=vr0=vr0dc8meaabaqaciaacaGaaeqabaqabeGadaaakeaaieqacuWF4baEgaqcamaaBaaaleaacqWGPbqAaeqaaaaa@2FC2@**w**^*t*^)_+ _    (5)

and (...)_+ _denotes the positive part of the quantity in the parenthesis, as above, *i.e*. equal to the quantity if it is greater than zero, and zero otherwise.

Since the capacity of the classifier increases with increasing norm of the weight vector, the parameter *C *also controls the tradeoff between the size of the margin and the capacity of the classifier.

Since the samples need to be represented only in the form of scalar products, this formulation can be extended to non-linear classifiers by the introduction of *kernels*. Kernels are the functional representation of scalar products in transformed space. It can be shown that such a transformation leaves the optimization problem unchanged except that the inner product ⟨**x**_1_, **x**_2_⟩ is replaced by the kernel *K*(**x**_1_, **x**_2_). In the case of very high (or infinite) dimensional transformed space, the kernel is usually easier to compute than doing the transformation followed by scalar product.

For Gaussian kernel SVM (also called a radial basis function kernel), the kernel is given by

*K*(**x**_1_, **x**_2_) = exp(-γ ||**x**_1 _- **x**_2_||^2^)     (6)

The spread of the kernel function is given by γ, which can be varied to adapt the kernel to the data. The larger the value of γ, the more peaked the corresponding transformations of the feature vectors are, and the higher the capacity of the classifier.

Peng *et al *use the Leave-One-Out-CV (LOOCV) error to tune the kernel parameters. LOOCV is a CV scheme where one sample is left out during each iteration. The average classification error obtained is an almost unbiased estimate of the true error. In (6), the training data is used to select an appropriate kernel (from linear, Gaussian and polynomial) and set of parameters that minimize the LOOCV error estimate. However no independent test set is used and only the final LOOCV error estimate on the training set is reported.

## Results

### Implementation

For each simulation we generated at least 1000 sets of 40 samples, of which 20 belonged to class 1 and the remaining 20 to class 2. Each sample was a vector of 6000 features (synthetic gene expressions). For some of the cases we used "null" data sets where no gene is differentially expressed between the two classes. For each gene, the population mean expressions in both classes were the same, namely zero. Thus none of the features are truly discriminatory between the two classes. We also used data generated from a "non-null" distribution for validating the nested CV approach. In this case, instead of using a "null distribution" (i.e. no difference in gene expression between the two classes), we simulated differential expression by fixing 10 genes (out of 6000) to have a population mean differential expression of 1 between the two classes. Samples from one class were drawn from a mean zero, unit variance, multivariate Normal distribution while samples for the second class had 10 genes with mean 1, unit variance Normal and the rest 5990 genes were of mean zero, unit variance Normal distribution.

### Shrunken centroids

This was implemented in MATLAB^™ ^(Ver. 6.5, The Mathworks). For this case we used the "null" dataset with no difference between the two classes. For each 40 sample dataset, we computed the 10-fold CV error for the Shrunken Centroid classifier for values of Δ between 0.01 and 1. The value of Δ with the minimum 10-fold CV error *CV*(Δ) was determined.

Δ^* ^= arg min (*CV*(Δ)

If two values of Δ had the same error, the larger value was chosen (this corresponded to smaller number of genes with non-zero weights).

To determine if the CV error estimate *CV*(Δ^*^) is a unbiased estimate of the true error for a classifier built using all the 40 samples with parameter value Δ^*^, we created a Shrunken centroid classifier using all 40 samples and parameter value of Δ^*^. We call this the *optimized Shrunken Centroids classifier*. This classifier was used to predict the class of 20000 samples created independently using the "null" data distribution. Since these test samples were not part of the training set, the mean error on them will give us the true error *TE*(Δ^*^).

This process was repeated for each simulated 40-sample dataset providing empirical distributions of *CV*(Δ^*^)and *TE*(Δ^*^).

### Support Vector Machine

The same type of analysis was performed for the SVM case. The "null" data distribution was used to create the synthetic data. For computational efficiency, we do not consider the complete algorithm used in (6). Instead of recursive feature elimination (RFE) for feature selection, we used the two sample t-statistics and selected the three features with the largest absolute t-statistic. In lieu of genetic algorithms (GA) as the search strategy, we used a simplified algorithm with a fixed Gaussian kernel. The classifier parameters tuned were *C *(trade off parameter in Eq. 4) and γ (kernel parameter). It must be noted that reducing the set of parameters over which one optimizes, as we have done here, may potentially reduce the amount of bias obtained.

The Leave-One-Out-CV (LOOCV) was used to compute the CV error estimate for each point on a grid of *C *and γ values (*C *was varied from 2^-5 ^to 2^15 ^and γ from 2^-15 ^to 2^3^). The CV error estimate was computed by leaving out each of the 40 samples in turn, selecting the 3 best features using the t-statistic on the remaining 39 samples and creating an SVM classifier using the fixed *C *and γ values. This classifier was used to predict the class of the left out sample. The average error on the 40 samples is a CV error estimate *CV*(*C*, γ) for the values of *C *and γ used. This was repeated for all of the grid values. We used the LIBSVM package developed by Chang and Lin [9] to develop the classifier.

Similar to the analysis on Shrunken Centroids, we find the value of parameters that minimize the CV error estimate

(C^*^, γ ^*^) = arg min (*CV*(*C*, γ))

To compute the true error, an SVM classifier with parameters C^* ^and γ ^* ^was built using all 40 samples, and the top 3 features based on all 40 samples. We call this the *optimized SVM classifier*. This classifier was used to predict the classes of a large test set of 20000 samples with the same distribution as the training set (*i.e*. the "null" distribution). The mean error on the test set gives us the true error *TE*(C^*^, γ ^*^).

### Nested CV with shrunken centroids and SVM

We evaluated the nested CV approach for the Shrunken Centroids classifier with Δ optimized using10-fold CV (the optimized Shrunken Centroids classifier). The "null" data distribution was used to generate the datasets. Finding the optimum value Δ^* ^of the classifier parameter was done exactly like described above, as was finding the true error *TE*(Δ^*^). The only thing that changes is how a new estimate of the true error is computed. Instead of using the CV error estimate *CV*(Δ^*^) for the optimal Δ, we used the *nested CV error estimate*. The nested CV error estimate is computed this way. One sample (out of the 40) was left out and the Δ for the Shrunken Centroids classifier was selected on the remaining 39 samples by minimizing the 10-fold CV. This is done exactly as above except that the wrapper algorithm is restricted to 39 samples, instead of 40. The wrapper algorithm determines an optimal value of Δ using the 39 samples and then creates a classifier with the same 39 samples and the optimal value selected based on these samples. This classifier is used to predict the class of the left out sample. This was done for each of the 40 samples, left out in turn. The average error over the samples is the nested CV error estimate *CV*_*nest*_(Δ^*^) for the optimized Shrunken Centroid classifier. Repeating this for several synthetic datasets gives us the empirical distribution of *CV*_*nest*_(Δ^*^) and *TE*(Δ^*^).

The nested CV approach was also evaluated for the optimized SVM classifier. Here we used the "non null" data distribution to create the training samples (40 samples) and the test samples (20000 samples). Nested CV is used to find the nested CV error estimate *CV*_*nest*_(C^*^, γ ^*^) of the SVM classifier optimized for parameters *C *and γ. The procedure is exactly the same as that described for optimized Shrunken Centroids above, except that the classifier training algorithm being evaluated is the optimized SVM classifier algorithm detailed earlier.

**Figure 1:**
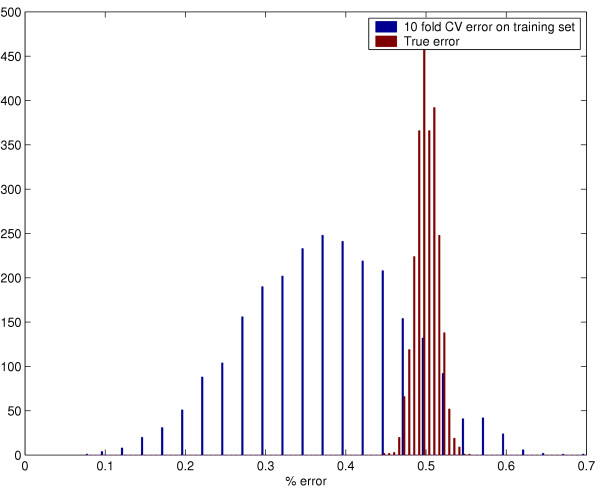
Distribution of the CV error estimate and the true error for optimized Shrunken Centroids.

**Figure 2:**
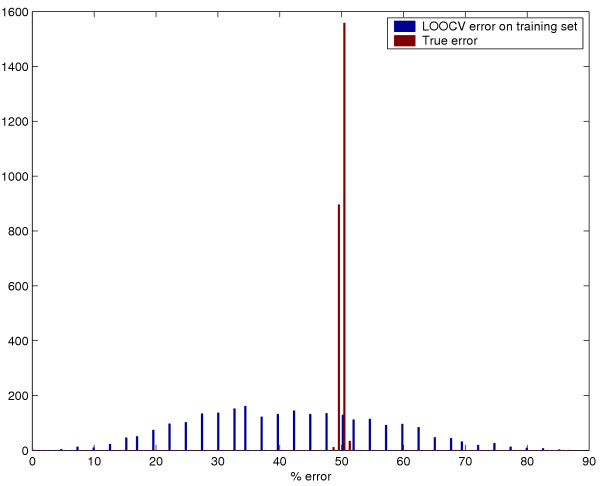
Distribution of the CV error estimate and the true error for optimized Support Vector Machine.

Fig 1 shows the empirical distributions of *CV*(Δ^*^), the CV error estimate for the optimal Δ and *TE*(Δ^*^), the true error for the optimal Δ for the optimized Shrunken Centroid classifier. Fig 2 shows the distributions for *CV*(C^*^, γ ^*^) and *TE*(C^*^, γ ^*^) for the optimized SVM classifier. In both plots, the CV error estimate is centered over means that are distinctly smaller than the mean true errors. Since both these simulations were done with "null" data, the true errors are centered on 50% while the CV error estimates have a lower mean. Numerical results are shown in Table 1. Even though the mean true error is 50% (*i.e*. equal to randomly choosing the classes), the CV error estimate on the training set averages 37.8% for the optimized Shrunken Centroid classifier and 41.7% for the optimized SVM classifier. On more than one-fifth of the random training datasets, the bias is more than 20% for the classifiers.

**Table 1:** Results.

**Classifier type**	**Percentage of times** ** training error<30%**	**Percentage of** ** times bias>20%**	**Mean CV error estimate** ** for optimized classifier**	**Mean true error of** ** optimized classifier**	**Mean bias**
Shrunken centroids on "null" data	18.5%	22.2%	37.8%	50.0%	-12.2%
SVM on "null" data	22.2%	25.3%	41.7%	50.0%	-8.3%

**Figure 3:**
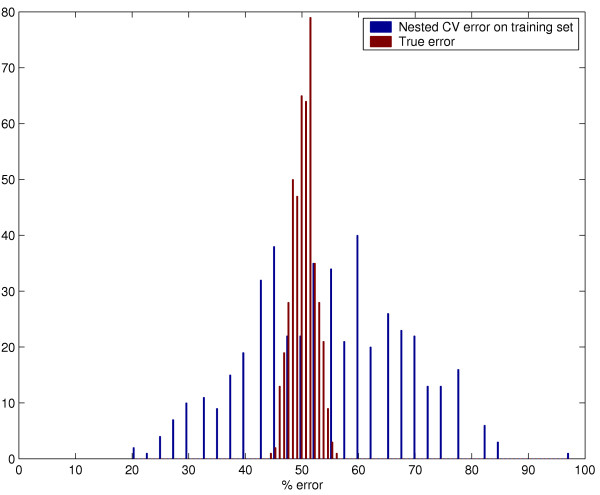
Distribution of the nested CV error estimate and true error for optimized Shrunken Centroids nested within a LOOCV loop.

Fig 3 shows the distributions for the nested CV error estimate *CV*_*nest*_(Δ^*^) and the true error *TE*(Δ^*^) for the optimized Shrunken Centroids. We obtain an almost unbiased estimate of the true error. The mean nested CV error estimate is a slight overestimate of the true error (54.2% compared to 50.0%), since the classifier used in each nested CV iteration is based on 39 samples, while the classifier used on the test set is trained on 40 samples.

**Figure 4:**
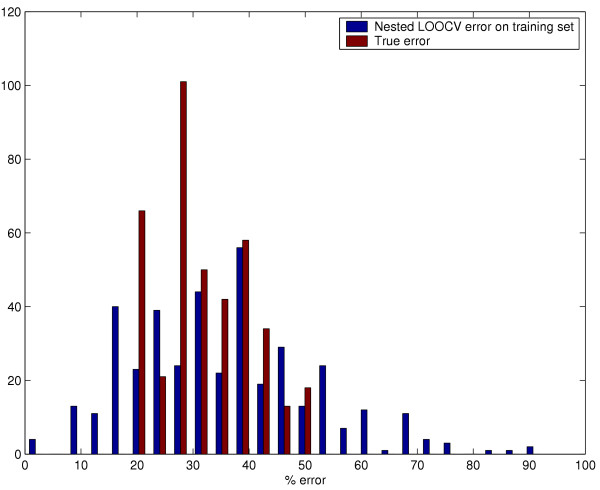
Distribution of the nested CV error estimate and true error for optimized SVM nested within a LOOCV loop.

Fig 4 shows the distribution of the nested CV error estimate *CV*_*nest*_(C^*^, γ ^*^) and the true error *TE*(C^*^, γ ^*^) for the optimized SVM algorithm on the "non null" data sets. We obtain a mean nested CV error estimate of 35.3% compared to the true error of 32.0%. The higher estimate of training error compared to test error can again be attributed to the lower number of samples being used (39 vs. 40) to create the classifier in the nested CV iteration.

## Conclusion

Our results demonstrate that although it is reasonable to optimize classifier parameters by minimizing cross validated error rates, the resulting minimum CV error estimate is not an unbiased estimate of the true error that can be expected from the final classifier on independent data. The difference between the CV error estimate and the true error can be greater than 20% more than one-fifth of the time which can be very significant in classification problems where the overall accuracy is not very high (such as predicting survival or response to treatment).

In our work, we observed this bias for two different resampling methods (10-fold CV and LOOCV). Will this bias, possibly with differing magnitudes, be present for other cross validation schemes? Since the bias is caused by the variability in the estimates of prediction error for different values of the tuning parameters it is a general phenomenon. This can be seen intuitively in the following simple explanation.

Assume that classifier parameter α can only take discrete values α_1_, α_2_, ..., α_*k *_and that the true prediction error *E *is independent of the value of the parameter. Thus all values of the parameter will give the same true error. However, due to the variability in the errors estimated by resampling, different parameter values will lead to different prediction error estimates. Let the error estimates be denoted by *e*_1_, *e*_2_, ..., *e*_*k*_. If we assume that the resampling method is median unbiased, it is easy to see that the probability that the minimum of the errors is lesser than *E*

Pr(min {*e*_2_, ..., *e*_*k*_} <*E*) = 1 - (12)K
 MathType@MTEF@5@5@+=feaafiart1ev1aaatCvAUfKttLearuWrP9MDH5MBPbIqV92AaeXatLxBI9gBaebbnrfifHhDYfgasaacH8akY=wiFfYdH8Gipec8Eeeu0xXdbba9frFj0=OqFfea0dXdd9vqai=hGuQ8kuc9pgc9s8qqaq=dirpe0xb9q8qiLsFr0=vr0=vr0dc8meaabaqaciaacaGaaeqabaqabeGadaaakeaadaqadaqaamaalaaabaGaeGymaedabaGaeGOmaidaaaGaayjkaiaawMcaamaaCaaaleqabaGaem4saSeaaaaa@3173@     (8)

This implies that for large *K*, there is a high probability that choosing the minimum resampled error will give a biased estimate of the true error.

Even if there is no parameter selection being done, any resampling method for estimating error is subject to some bias and variance (11). We have mentioned above the bias of the LOOCV method (over-estimating the true error) that occurs because a subset of training samples is used to create the classifier in the CV loop while the final classifier is created using all the samples. We call this the *inherent bias*. However, as we have shown in our results, when additional parameter selection is being done, the variance of the estimates results in an additional *parameter selection bias*. The parameter selection bias adds to the inherent bias to form the total bias of the resampling procedure. It must be noted that inherent bias can be either negative or positive (*i.e*. the error estimate can be lower or higher than the true error). The parameter selection bias, on the other hand, is always negative.

The parameter selection bias is smaller for resampling methods with smaller variances, *e.g*. .632 Bootstrap [10] and Bootstrap CV [11]. However, low variance methods can also have a large inherent bias [12] (either positive or negative). The total bias (*i.e*. the sum of inherent bias and parameter selection bias) is what must be kept in mind when interpreting error estimates. If the inherent bias is positive, the parameter selection bias will subtract from it and possibly bring the error estimate closer to the true error. However, if the inherent bias is negative, the parameter selection bias will exacerbate it.

Cross-validation can be a useful method for selecting tuning parameters for a classifier, but the generalization error for the resulting classifier should be estimated correctly. This can be accomplished by a large validation set that is independent of data used for parameter tuning. However this requires a large number of samples that can be split into training and validation sets. Another way, used in this paper and illustrated by the method of Izuka *et al*. [7], is to leave out some samples that are not used in the CV tuning of the parameters. A tuned classifier is then developed on the reduced training set and tested on the left out samples; this procedure is repeated for several sets of left out samples. This is effectively two nested CV loops; the outer loop estimates the generalization error while the inner CV is used for tuning the parameters. If the number of samples left out at each step of the outer loop is not too large, this gives an almost unbiased estimate of the true error.
